# Application of Design of Experiment for Floating Drug Delivery of Tapentadol Hydrochloride

**DOI:** 10.1155/2013/625729

**Published:** 2013-06-26

**Authors:** Swati C. Jagdale, Somnath Patil, Bhanudas S. Kuchekar

**Affiliations:** ^1^MAEER's Maharashtra Institute of Pharmacy, MIT Campus, Paud Road, Kothrud, Pune, Maharashtra 411038, India; ^2^Department of Pharmaceutics, MAEER's Maharashtra Institute of Pharmacy, MIT Campus, S.No. 124, Kothrud, Pune, Maharashtra 411038, India

## Abstract

The aim of the present study was to apply design of experiment (DOE) to optimize floating drug delivery of tapentadol hydrochloride. Tapentadol hydrochloride is a synthetic opioid used as a centrally acting analgesic and effective in both experimental and clinical pain. The half-life of the drug is about 4 hours and oral dose is 50 to 250 mg twice a day. For optimization 3^2^ full factorial design was employed for formulation of tapentadol hydrochloride tablets. Sodium bicarbonate was incorporated as a gas-generating agent. Combination of polymers Xanthan gum and Locust bean gum was used to achieve controlled release effect. The concentration of polymers was considered as the independent variables and dependent variables were floating lag time and swelling index of the tablets. From the factorial batches, it was observed that formulation containing combination of 20% sodium bicarbonate and 10% citric acid shows optimum floating ability whereas the formulation containing 20% Xanthan gum and 28% Locust bean gum shows optimum sustained drug release pattern with adequate floating.

## 1. Introduction

Oral administration is the most convenient and preferred means of any drug delivery to the systematic circulation. Oral controlled release drug delivery has recently been of increasing interest in pharmaceutical field to achieve improved therapeutic advantages, such as ease of dosing administration, patient compliance, and flexibility in formulation. Drugs that are easily absorbed from gastrointestinal tract (GIT) and have short half-lives are eliminated quickly from the systemic circulation. Frequent dosing of these drugs is required to achieve suitable therapeutic activity [1]. Various oral controlled delivery systems have been designed which can overcome the problems of unpredictable gastric emptying due to physiological problems and presence of food and also release the drug to maintain its plasma concentration for a longer period of time. This has led to the development of oral gastro-retentive dosage forms. This dosage form improves bioavailability, therapeutic efficacy and may allow a reduction in the dose because of steady therapeutic levels of drug [2]. Gastro-retentive drug delivery system is commonly employed for the formulation of controlled release drug delivery in stomach. Among the various approaches for formulating the gastro-retentive drug delivery system, floating system is one of the most commonly used. Floating system involves the gas-forming agent that helps in keeping the formulation in buoyant state and hence avoids its passage through stomach facilitating the controlled release of drug from formulation [3]. Tapentadol is a centrally acting analgesic with a low affinity for opioid receptors. Tapentadol is a synthetic codeine analogue that is a weak mu-opioid receptor agonist. Part of its analgesic effect is produced by inhibition of uptake of norepinephrine and 5-hydroxytryptamine. In the treatment of mild-to-moderate pain, tapentadol is as effective as morphine or meperidine. The half-life of the drug is about 4 hours and the approximate equianalgesic dose is 50–250 mg twice a day [4]. Tapentadol is available in market as immediate release tablet for the treatment of acute pain [5]. Hence for the treatment of chronic and moderate pain a floating controlled release formulation was prepared. Tapentadol hydrochloride is freely soluble in water; hence release-retarding polymers such as Xanthan gum and Locust bean gum play an important role in controlling the release of tapentadol from the formulation. The floating of the formulation can be achieved by incorporating gas-generating agent such as sodium bicarbonate and citric acid.

## 2. Materials and Methods

### 2.1. Materials

Tapentadol hydrochloride was provided as a gift sample from JCPL, Jalgaon, and Xanthan gum and Locust bean gum were obtained as a gift sample from Vapi Care Pharma Pvt Ltd., Vapi. Other excipients as sodium bicarbonate, mannitol, magnesium stearate, citric acid, and chemicals were of analytical grade and purchased from Pure Chem. Laboratories, Pune.

### 2.2. Methods

#### 2.2.1. Experimental Design

A 3^2^ level full-factorial design includes 9 full factorial design points; according to the model, total 9 experiments were conducted. This design involves dependent variables and independent or controlled variables *X*1 and *X*2. In the present study, experiment was conducted considering concentration of Xanthan gum and Locust bean gum as independent variables. The dependent variables were *Z*1, percent drug release after 8 hours, *Z*2; hardness, *Z*3; swelling index, and *Z*4; floating lag time.

#### 2.2.2. Preparation of Tapentadol HCl Tablets

The trial batches were prepared using various concentrations of the sodium bicarbonate and polymers. The concentration of polymers for the factorial design was finalized based on the evaluation of trial batches. In preliminary study, sodium bicarbonate was used in range of 15–20% concentration as floating agent. Citric acid (10%) was used in combination with sodium bicarbonate in all batches. During trial, tablets containing 30–40% of Xanthan gum alone as well as 50–60% of Locust bean gum alone were prepared and evaluated for floating lag time and drug release pattern. The tablets of trial batches were prepared by direct compression method using 8-station rotary press tablet compression machine using the formulae as shown in Table 1.

From the trial batches, design of experiment (DOE) was applied to optimize the formula. 3^2^ full factorial design (Table 2) was employed to optimize the concentration of Xanthan gum and Locust bean gum.

Depending on the results of the trial batches, tablets were prepared using the factorial design as shown in Table 2. All the batches were evaluated for various parameters and the formulations showing optimized results were found out. The concentration of sodium bicarbonate (20%) and citric acid (10%) was finalized and the factorial design was applied to find out the optimized formulation containing Xanthan gum and Locust bean gum. Magnesium stearate (5 mg) was used in each formulation for the purpose of lubrication. The polymers were added in the formulation as specified in the factorial design. All the ingredients were uniformly mixed in powder form in the polythene bag and the resultant powder mixture was compressed in the 8-station rotary press tablet compression machine. The tablets were round and flat with an average diameter of 9 mm and a thickness of 5 mm.

#### 2.2.3. Evaluation of Powder Blend

The powder blend used for preparation of tablets was evaluated for angle of repose and compressibility index.

For angle of repose 10 gm of powder was passed through funnel and the pile was formed. The height and weight of the pile were measured and the angle of repose was calculated by using the following formula:

(1) $$\begin{array}{c} \text{Angle}\;\text{of}\;\text{repose}\;\left(\theta \right)=\tan^{-1}\left(\frac{\text{height}}{\text{radius}}\right). \end{array}$$

The angle of repose less than 30° usually indicates a free-flowing material and more than 40° suggests a poorly flowing material [6].

Carr's compressibility index was calculated by calculating the tapped and bulk density using 100 mL measuring cylinder. Compressibility is calculated by the following formula:

(2) $$\begin{array}{c} \text{Carr's}\;\text{compressibility}\;\text{index}=\frac{\left(\text{TBDLBD}\right)}{\text{TBD}}\times 100, \end{array}$$

where TBD is tapped bulk density and LBD is loose bulk density. Carr's index greater than 25 is considered to be an indication of poor flow ability and below 15 of excellent flow ability.

#### 2.2.4. Evaluation of Tablets

All the formulations were evaluated for various parameters such as hardness, friability, weight variation, % drug content, buoyancy lag time, swelling index, *in vitro* drug release, release experiments, and IR spectroscopy [7].

Hardness of tablets was determined using the Monsanto hardness tester [7].

For each formulation, the friability of 20 tablets was determined using the Roche friabilator. In this test tablets were subject to the combined effect of shock abrasion by utilizing a plastic chamber which revolves at a speed of 25 rpm, dropping the tablets to a distance of 6 inches in each revolution. A sample of preweighted 20 tablets was placed in the Roche friabilator which was then operated for 100 revolutions, that is, 4 mins. The tablets were then dusted and reweighed. Percent friability (%*F*) was calculated as follows:

(3) $$\begin{array}{c} \%F=\left(\frac{\text{loss}\;\text{in}\;\text{weight}}{\text{initial}\;\text{weight}}\right)\times 100. \end{array}$$

Conventional compressed tablets that lose less than 0.5 to 1.0% of their weight are generally considered acceptable [8].

Thickness of all tablets was measured using a vernier calliper [7].

The weight of 20 tablets was taken on electronic balance and the weight variation was calculated.

The weight variation tolerance allowed for tablet weighing 324 mg and more is 5% as per IP [9].

For drug content ten milligrams of the tablet powder was added to 10 mL of 0.1 N HCL and drug solution was filtered through the Whatman paper number 1. The sample was analyzed for drug content by UV spectrophotometer (Varian Cary 100) at 272 nm after suitable dilutions. 

*In vitro* buoyancy was determined by buoyancy lag time. The tablets were placed in a 100 mL beaker containing 0.1 N HCl. The time required for the tablet to rise to the surface and float was determined as floating lag time [10].

The swelling index of the tablets was calculated in order to find out the swelling ability of the tablets. For calculating the swelling index, the previously weighed tablets were placed in the 100 mL beaker containing 0.1 N HCl. The tablets were removed at the time interval of 1 hr for 8 hours and weighed. The swelling index of the tablets can be measured by studying its dimensional changes, weight gain, or water uptake. Hence swelling index was calculated by the following formula:

(4) $$\begin{array}{c} \text{Swelling}\;\text{index}=\frac{\left(W_{t}-W_{o}\right)}{W_{o}}\times 100, \end{array}$$

where *W*_*t*_ is the final weight of tablets at time “*t*” and *W*_*o*_ is the initial weight of tablets [11].

The release rate of tapentadol HCl from floating tablets was determined using United States Pharmacopeia (USP) dissolution testing apparatus 2 (paddle method). The dissolution test was performed using 900 mL of 0.1 N HCl, at 37 ± 0.5°C and 75 rpm. A sample (5 mL) of the solution was withdrawn from the dissolution apparatus hourly for 8 hours, and the samples were replaced with fresh dissolution medium. The samples were filtered through a 0.45 *μ* membrane filter and diluted to a suitable concentration with 0.1 N HCl. Absorbance of these solutions was measured at 272 nm using double beam UV spectroscopy [12].

#### 2.2.5. Kinetic Modeling

The dissolution profile of all the batches was fitted to zero-order, first-order, matrix, Hixson-Crowell, Korsmeyer, and Peppas to ascertain the kinetic modeling of drug release. The kinetic modeling was found out by employing the PCP Disso v3 software [13].

#### 2.2.6. Infrared (IR) Spectroscopy

The drug excipient compatibility and the drug polymer interaction were detected by the IR spectroscopic studies. The polymer—polymer compatibility—is also found out by the IR spectroscopic studies.

#### 2.2.7. Optimization Data Analysis and Validation of Optimization Model

Various RSM computations for the current optimization study were performed employing Design Expert software (Version 8.0.2, Stat-Ease Inc., Minneapolis, MN, USA). Polynomial models including interaction and quadratic terms were generated for all the response variables using multiple linear regression analysis (MLRA) approach. The general form of the MLRA model is represented as in (5):

(5) Y=β0+β1X1+β2X2 +β3X1X2+β4X12+β5X22,

where *β*0 is the intercept representing the arithmetic average of all quantitative outcomes of 9 runs; *β*1 to *β*5 are the coefficients computed from the observed experimental values of *Y*; and *X*1 and *X*2 are the coded levels of the independent variable(s). The terms *X*1*X*2 and *Xi*2 (*i* = 1 to 2) represent the interaction and quadratic terms, respectively. Statistical validity of the polynomials was established on the basis of ANOVA provision in the design expert software. Subsequently, the feasibility and grid searches were performed to locate the composition of optimum formulations. Also, the 3D response surface graphs and 2D contour plots were constructed in MS excel environment using the output files generated by the design expert software. Eight optimum checkpoints were selected by intensive grid search, performed over the entire experimental domain, to validate the chosen experimental design and polynomial equations. The formulations corresponding to these checkpoints were prepared and evaluated for various response properties. Subsequently, the resultant experimental data of response properties were quantitatively compared with that of the predicted values. Also, linear regression plots between observed and predicted values of the response properties were drawn using MS-excel, forcing the line through origin [14].

## 3. Results and Discussion

The various trial batches were conducted to optimize the concentration of NaHCO_3_ (Table 1). Trial batches were evaluated for parameters such as buoyancy lag time and % drug release after 8 hours. Formulations containing 15% and 20% of sodium bicarbonate alone failed to float whereas formulation containing 20% sodium bicarbonate along with 10% citric acid showed good floating. 

It was observed that formulations containing 150 mg and 180 mg of Locust bean gum alone showed immediate floating but formulations dissolved within 2 hours, while formulations containing 90 mg and 120 mg of Xanthan gum alone showed floating within 5 min and retardation of drug release for more than 8 hours. Hence combination of these two polymers was used to get optimum floating ability and drug release. Formulation containing Locust bean gum (70 mg) and Xanthan gum (50 mg) in combination showed optimum floating and release pattern (Table 3).

### 3.1. Evaluation of Powder Blend (F1–F9)

Angle of repose of all the powder blends was obtained within the range of 20–30° indicating good ability to flow [15]. Carr's compressibility index of all the powder blends was obtained below 10 indicating good compressing capacity of the powder blend. 

### 3.2. Evaluation of Tablets

Hardness of the formulations F1–F9 was observed within the range of 6.9–8.8 kg/cm^2^ as shown in Table 4. Friability of the tablets was observed below 0.30% for all batches which was in the acceptable limit. The thickness of all the tablets was found within the range of 5 ± 2 mm.

The weight of all the tablets was found within the range of 250 mg ± 5 mg. The range of % drug content of the formulations F1–F9 was found between 97.25 and 102.67. The *in vitro* buoyancy study showed the good floating ability of the tablets as shown in the Table 4. Buoyancy lag time indicates the time required for the formulation to float in the medium. From Table 4, it was observed that formulations F6 and F9 show comparatively more floating time as compared to other formulations. It was further observed that formulation F2 shows less floating time than others. This indicates that higher concentration of NaHCO_3_ affects the release pattern of drug from formulation whereas lower concentration (less than 20%) alone fails to float within a minute.

From evaluation of formulations F1–F9, it was observed that there is linear relationship between swelling index and concentration of polymers. Maximum swelling index was observed with F9 containing maximum concentration of both the polymers. From the swelling index study of all the batches, it was observed that the increase in the concentration of polymers increases the swelling property of the tablets as shown in Table 4. Further the formulation containing optimized swelling index was obtained. From the formulation batches, it was observed that the formulations F9 showed maximum swelling index.

### 3.3. *In vitro *Dissolution Studies

**Figure 1:**
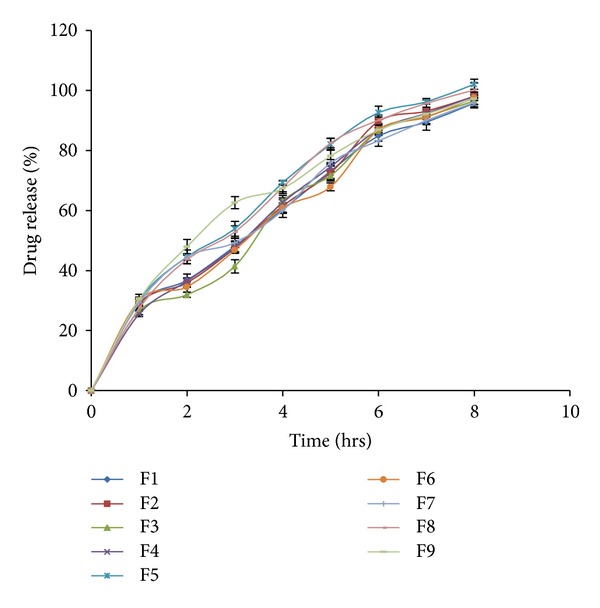
% Drug release profile of drug from formulations containing Xanthan gum and Locust bean gum.

Locust bean gum has low gelling and matrix forming property than Xanthan gum. Hence Locust bean gum alone cannot be used as matrix polymer in the formulation of Matrix tablet. Xanthan gum alone as well as in combination with other gums is good matrix polymer to formulate controlled-release tablets. Locust bean gum (50–60%) alone fails to retard drug release. Xanthan gum (30–40%) alone gives good retardation of drug release for extended period of time. The drug release patterns from all the formulations are shown in Table 4. The percent drug release after 8 hours is as shown in Figure 1. 

The drug release profile of formulations F1–F9 indicates that as the concentration of polymers increases, the drug release decreases. From the comparison of release profiles of all the batches, it was observed that the formulations containing combination of polymers show retardation of drug release to a greater extent than formulations containing single polymer. The batches F5, F8, and F9 compliy with standards for drug release as mentioned for Modified-release tablet in USP29 [16]. 

From the *in vitro* dissolution studies and the response surface curves, it was observed that the drug release pattern was influenced by the variation in the concentration of polymers. Batches F5, F8, and F9 show optimum drug release profiles but batch F9 fails to float within 1 min. As compared with batch F8, batch F5 has higher swelling index as well as optimum FLT and drug release. The infrared studies show that there is no interaction between the excipient and drug that can affect the efficacy of drug. 

### 3.4. Kinetic Modeling

From the kinetic modeling study (Table 5), it was observed that most of the formulations showed Peppas as the best fitting model. The Hixson-Crowell and Matrix models were also observed as the best fitting in some formulations. The equations of the best fitting model are as follows:

 The Korsmeyer and Peppas model:
  (6) $$\begin{array}{c} F=ktn. \end{array}$$
  The Hixson and Crowell powder dissolution method:
  (7) $$\begin{array}{c} F=100\left(1-\left(1-kt\right)3\right). \end{array}$$
 The Higuchi Matrix:
  (8) $$\begin{array}{c} F=k\sqrt{t}, \end{array}$$

where *F* is the fraction of drug release, *k* is the release constant, *t* is the time, and *n* is diffusion coefficient. From the *in vitro* dissolution studies and the response surface curves, it was observed that the drug release pattern was influenced by the variation in the concentration of polymers. When kinetic modeling was fitted to batch F5 Peppas type of release pattern shows fair linearity with regression value of 0.6239. This indicates that the release mechanism is not well known or more than one type of release phenomena is involved as Fickian diffusion (Higuchi Matrix), anomalous transport, and zero-order release. None of the formulations fit into zero-order equation indicating that the dissolution rate of drug is independent of the amount of drug available for dissolution and diffusion from the tablets.

### 3.5. RSM Optimization

Equations of the formulations containing Xanthan gum and Locust bean gum (F1–F9): mathematical modeling and mathematical relationships generated using MLRA for the studied response variables are expressed as in (9) and (10):

(9) Floating  lag  time=+58.29+0.67∗A+11.17∗B +6.50∗A∗B−5.57∗A2 +17.93∗B2,

(10) Swelling  index=+293.02 +0.25∗A+10.17∗B,

where *A* and *B* represent the effect of variables, that is, concentration of Xanthan gum, and Locust bean gum respectively. All the polynomial equations were found to be statistically significant (*P* < 0.01), as determined using ANOVA, as per the provision of Design Expert software. The model *F*-value of 14.95 in (9) implies the model is significant. Values of “Prob > *F*” less than 0.0500 indicate model terms are significant. In this case *B*, *B* ^2^ are significant model terms. The model *F*-value of 10.09 implies the model is significant. Values of  “Prob > *F*” less than 0.0500 indicate model terms are significant. In this case *B* are significant model terms (10). The polynomial equations comprise the coefficients for intercept, first-order main effects, interaction terms, and higher-order effects. The sign and magnitude of the main effects signify the relative influence of each factor on the response. 

### 3.6. Studies of Floating Lag Time by Response Surface Methodology

**Figure 2:**
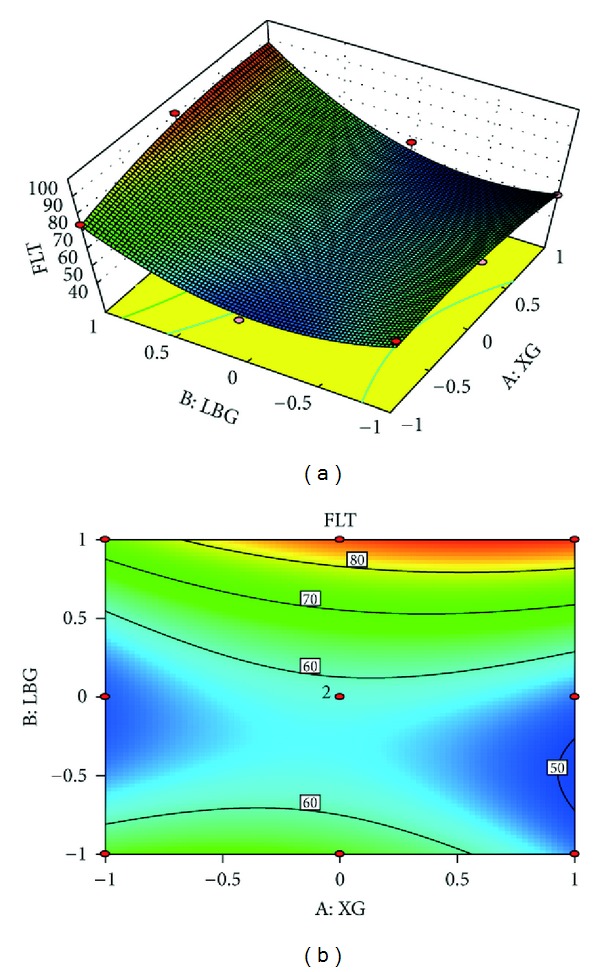
(a) Response surface plot showing the influence of Locust bean gum and Xanthan gum on floating lag time and (b) corresponding contour plot showing the relationship between various levels of two polymers.

Figure 2 shows the combined effect of polymers on Floating lag time. The plots indicates that optimum floating lag time was obtained with formulation F5.

### 3.7. Study of Swelling Index by Response Surface Methodology

**Figure 3:**
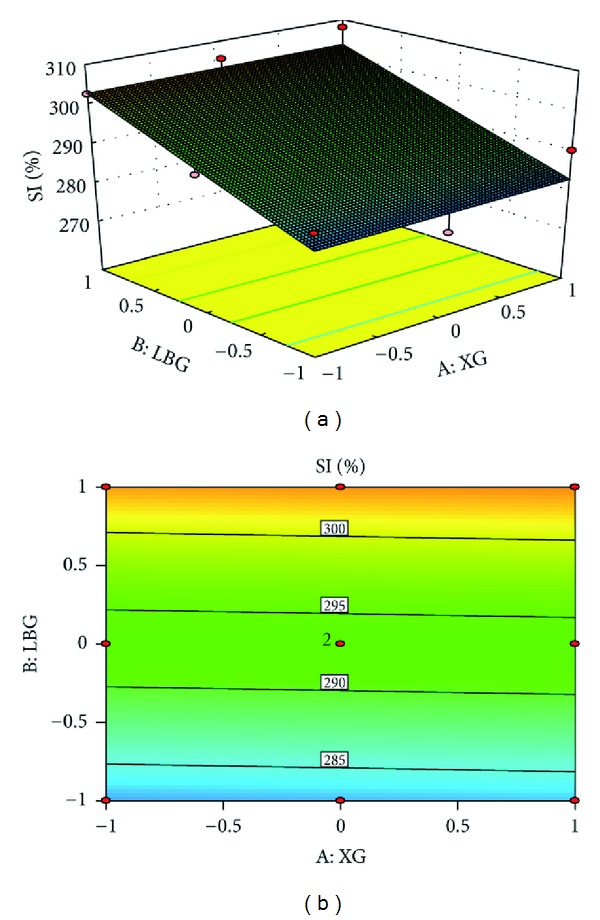
(a) Response surface plot showing the influence of Locust bean gum and Xanthan gum on swelling index and (b) corresponding contour plot showing the relationship between Locust bean gum and Xanthan gum.

Figure 3 shows the profound effect of concentration of the Locust bean gum and Xanthan gum on the swelling index of the formulation. The counter plot clearly indicates that the swelling index is increased with increase in concentration of Locust bean gum and Xanthan gum (10).

From the formulations containing Locust bean gum and Xanthan gum, it was observed that the optimized floating and swelling index was obtained with the formulation F5. From the swelling index study of F1–F9, it was observed that formulations containing high concentration of Locust bean gum and Xanthan gum show more swelling index. Maximum swelling index was observed with F9 containing maximum concentration of both the polymers.

### 3.8. Infrared (IR) Spectroscopic Study of the Formulation

**Figure 4:**
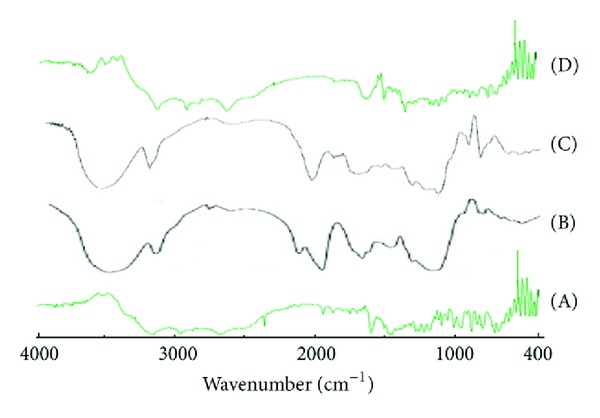
IR spectroscopic study of drug, polymer, and formulations. (A) Tapentadol, (B) Xanthan gum, (C) Locust bean gum, and (D) formulation (F5).

Figure 4 shows the infrared spectroscopic scan of tapentadol hydrochloride mixed with KBr. The IR scan shows prominent peaks for the various active groups such as 3554 cm^−1^ corresponding to the N–H stretch in the tertiary amino group, 1457 cm^−1^ corresponding to the C–O stretch between phenolic C and O group.

The formulations containing the polymers showed all the prominent peaks of tapentadol HCl with no change in the intensity of the peaks. This indicates that there is no interaction between the excipient and drug that can affect the efficacy of drug.

### 3.9. Stability Study

There was no significant difference in floating time, % drug content, and amount of tapentadol hydrochloride released from F5 after storing for 6 months at normal conditions and for 3 months at 40°C temperature, 75% relative humidity.

## 4. Conclusion

Modified drug release attained in the current study indicates that the matrix tablets of tapentadol hydrochloride, prepared using various polymers, can successfully be employed as a once-a-day oral controlled release drug delivery system. High floating ability of the formulation is likely to increase its GI residence time and, eventually, improve the extent of bioavailability. However, appropriate balancing between various levels of the polymers and floating agent is imperative to acquire proper controlled release and flotation of the formulation. High degree of prognosis obtained using response surface methodology indicates that a 3^2^ factorial design is quite efficient in optimizing drug delivery systems that exhibit nonlinearity in response(s). Formulation F5 shows good *in vitro *gastro-retentive floating drug delivery of tapentadol HCl. 

## Figures and Tables

**Table 1:** Tablet formulations for preliminary trials.

Sr. No.	Ingredients	D1	D2	D3	D4	D5	D6	D7	D8	D9	D10	D11	D12
1	Drug	50	50	50	50	50	50	50	50	50	50	50	50
2	Sod. bicarbonate	60	60	60	70	60	40	50	50	50	50	50	50
3	Citric acid	30	30	30	40	30	20	25	25	25	25	25	25
4	Xanthan gum	—	40	40	40	70	—	—	—	30	40	50	50
5	Locust bean gum	150	110	100	90	80	180	180	100	70	60	50	70
6	Mannitol	5	5	15	5	5	5	—	20	20	20	20	—
7	Mg. stearate	5	5	5	5	5	5	5	5	5	5	5	5

	Total wt. (mg)	300	300	300	300	300	300	310	250	250	250	250	250

All the weights are in mg.

**Table 2:** 3^2^ Full factorial design for the preparation of batches.

Formulation no.	Coded levels	
	Variable 1	Variable 2
I	−1	−1
II	−1	0
III	−1	+1
IV	0	−1
V	0	0
VI	0	+1
VII	+1	−1
VIII	+1	0
IX	+1	+1

**Table 3:** Levels of investigated variables.

Variables used	Coded levels		
	−1	0	+1
Xanthan gum (mg)	40	50	60
Locust bean gum (mg)	60	70	80

**Table 4:** Evaluation results of formulations F1–F9.

Formulation no.	% Drug release within 8 hrs.	% Drug content	Swelling index	Buoyancy lag time (sec.)	Hardness (kg/cm^2^)
F1	95.8	98.60	286.7	69	7.8
F2	98.01	99.24	291.1	48	8.1
F3	96.48	97.89	302.7	75	8.6
F4	98.04	99.09	277.9	62	8.0
F5	102.05	101.80	292.2	58	6.9
F6	97.57	98.93	305.4	91	7.3
F7	95.96	100.56	290.4	53	8.6
F8	100.14	102.37	283.7	58	8.1
F9	97.22	101.46	307.9	85	8.8

**Table 5:** Kinetic modeling of formulations (F1–F9).

Batch	*n*	*k*	Best fitting model
F1	0.6321	25.9854	Peppas
F2	0.6582	25.3902	Peppas
F3	0.7102	22.5277	Hixon-Crowel
F4	0.7008	23.6991	Peppas
F5	0.6239	29.1835	Peppas
F6	0.6374	25.6453	Peppas
F7	0.5926	28.2604	Peppas
F8	0.6563	27.2530	Peppas
F9	0.5641	31.6038	Matrix
